# Myocardial clearance of technetium-99m-teboroxime in reperfused injured canine myocardium

**DOI:** 10.1186/s13550-014-0042-6

**Published:** 2014-08-01

**Authors:** David R Okada, Gerald Johnson, Robert D Okada

**Affiliations:** 1 Brigham and Women’s Hospital, Harvard Medical School, Boston 02446, MA, USA; 2 University of Tulsa, Tulsa 74104, OK, USA; 3 University of Oklahoma Health Sciences Center, 6208 S. Oswego Ave, Tulsa 74136, OK, USA

**Keywords:** Teboroxime, Ischemia, Reperfusion, SPECT

## Abstract

**Background:**

Recent technical developments using solid-state technology have enabled rapid image acquisition with single photon emission computed tomography (SPECT) and have led to a renewed interest in technetium-99m-teboroxime (Tc-99m-teboroxime) as a myocardial imaging agent. Tc-99m-teboroxime has demonstrated high myocardial extraction, linear myocardial uptake relative to flow even at high flow rates, rapid uptake and clearance kinetics, and differential clearance in the setting of ischemia. However, the myocardial clearance kinetics of Tc-99m-teboroxime in a model of myocardial injury has not been previously reported. Thus, the purposes of this study were to use a canine model of ischemia-reperfusion to (1) compare Tc-99m-teboroxime clearance kinetics in normal and ischemic-reperfused myocardium and (2) assess the utility of Tc-99m-teboroxime clearance kinetics in determining the severity of injury following ischemia-reperfusion.

**Methods:**

Thirteen dogs underwent left circumflex coronary artery (LCx) occlusion for either 30 min (IR30, *n* = 6) or 120 min (IR120, *n* = 7), followed by reperfusion, and finally Tc-99m-teboroxime administration 120 min after reperfusion. Microsphere blood flows were determined at baseline, during occlusion, after reperfusion, and before euthanasia. Post-mortem, area at risk was determined using Evans blue dye, and viability was determined using triphenytetrazolium chloride (TTC) staining. The hearts were then subdivided into 24 pieces and Tc-99m activity was measured in a well counter.

**Results:**

TTC-determined infarct area as a percentage of total left ventricular myocardium was 1.1% ± 0.3% for the IR30 group and 7.5% ± 2.9% for the IR120 group (*p* < 0.05). During coronary occlusion, both the IR30 and IR120 groups demonstrated decreases in percent wall thickening in the ischemia-reperfusion zone (IRZ) as compared with the normal zone (NZ). In the IR30 group, percent wall thickening in the IRZ recovered during the reperfusion phase as compared with the NZ. In the IR120 group, percent wall thickening in the IRZ remained depressed during the reperfusion phase and through the end of the experiment as compared with the NZ. Final Tc-99m-teboroxime myocardial IRZ/NZ activity ratio was 0.94 ± 0.01 for the IR30 group, compared to 0.80 ± 0.01 for the IR120 group (*p* < 0.05).

**Conclusions:**

Tc-99m-teboroxime demonstrates moderate differential clearance in a model of severe injury with 120 min of ischemia-reperfusion, but only minimal differential clearance in a model of mild injury with 30 min of ischemia-reperfusion. Thus, Tc-99m-teboroxime clearance kinetics may be helpful in differentiating normal and minimally injured from severely injured myocardium.

## Background

Technetium-99m-teboroxime (Tc-99m-teboroxime) is a myocardial imaging agent that was previously available for clinical myocardial perfusion imaging. However, image quality was significantly compromised by the relatively rapid myocardial clearance kinetics, and it was subsequently withdrawn from the market. Recently, the development of solid-state single photon emission computed tomography (SPECT) camera technology has provided for faster acquisition capabilities and has led to a renewed interest in Tc-99m-teboroxime, which is again available for investigational use [1],[2].

Tc-99m-teboroxime has several unique and favorable properties compared with other available agents. Tc-99m-teboroxime demonstrates high myocardial extraction, an excellent flow to uptake relationship even at high flow rates [3], and differential clearance both in animal models of ischemia [4]–[6] and in patients with ischemia [7]–[10]. However, little is known with regard to the kinetic behavior of Tc-99m-teboroxime in models of left ventricular injury.

Tc-99m-teboroxime localizes primarily on the myocyte membrane in cultured myocytes [11] and demonstrates accelerated clearance following myocyte membrane injury due to treatment with Triton-X100 in isolated rat hearts [12]. Furthermore, Tc-99m-teboroxime requires viable myocytes for retention [13]. Therefore, we hypothesized that myocardial injury due to ischemia followed by reperfusion would lead to more rapid Tc-99m-teboroxime myocardial clearance, which would be proportional to the degree of myocyte injury.

Accordingly, the purposes of this study were to use a canine model of ischemia-reperfusion to (1) compare Tc-99m-teboroxime clearance kinetics in normal and ischemic-reperfused injured myocardium and (2) assess the utility of Tc-99m-teboroxime clearance kinetics in determining the severity of injury following ischemia-reperfusion.

## Methods

All experimental animals were handled in accordance with the guiding principles of the American Physiological Society and by the Institutional Animal Care and Use Committee at our institution.

### Surgical model

Thirteen adult mongrel dogs (mean weight 20.2 kg, range 15 to 24 kg) were anesthetized with sodium pentobarbital (26 mg/kg). Supplemental anesthetic was administered throughout the experiment as necessary. The dogs were intubated and placed on a respirator (Harvard Apparatus, South Natick, MA, USA) with 95% oxygen. Vinyl catheters were inserted into both femoral arteries and the carotid artery to monitor arterial pressure, to provide a site for microsphere reference blood withdrawal, and to obtain specimens of blood for the determination of arterial pH, pCO_2_, and pO_2_. Adjustments were made as necessary to maintain these parameters within a normal physiologic range (pH = 7.35 to 7.45 and pCO_2_ = 30 to 40 mmHg). Arterial pO_2_ was maintained above 100 mmHg throughout the experiment. Vinyl catheters were also inserted into both femoral veins as sites for infusion of supplemental anesthetic and fluids as required.

The heart was exposed via a left thoracotomy at the fifth intercostal space and suspended in a pericardial cradle. A vinyl catheter was inserted into the left atrial appendage to monitor left atrial pressure and provide a site for injection of radiolabeled microspheres. A Swan-Ganz thermodilution catheter was inserted into the left jugular vein and passed through the right side of the heart until its tip rested in the pulmonary artery. This catheter was subsequently used for the measurement of cardiac output.

The left circumflex (LCx) coronary artery was then carefully dissected free near the origin, and a 2.0 to 2.5 mm electromagnetic flow probe (SP2202 Statham Blood Flowmeter, Gould Electronics, Dallas, TX, USA) was placed around the artery, so that LCx blood flow could be continuously monitored throughout the procedure. A snare occluder was loosely positioned distal to the electromagnetic flow probe. A stiff 22-gauge, 8-in. catheter (Deseret Medical, Inc., Sandy, Utah) was inserted retrograde into a small branch of the LCx for monitoring pressure distal to the snare occluder.

Mean and phasic systemic arterial pressure, left atrial pressure, and distal LCx pressure were continuously monitored throughout the protocol using pressure transducers (Statham P23, Gould Electronics). Lead II of the surface electrocardiogram was continuously monitored throughout the protocol on an 8 channel strip chart recorder (Model 2800s, Gould Electronics).

Two pairs of ultrasonic dimension crystals were placed on the endocardial and epicardial surfaces of the left ventricle in order to record regional wall thickness and function. One pair was placed in the zone of the myocardium perfused by LCx that would eventually become the ischemia-reperfusion zone (IRZ), and the other pair was placed in the zone perfused by the left anterior descending coronary artery (LAD) that served as the normal zone (NZ). The pinger of each pair of crystals was located on the epicardial surface, and the receiver was located on the endocardial surface. The crystals were connected to a 4 channel sonomicrometer (Model 120, Triton Technology, San Diego, CA, USA).

### Preparation of Tc-99m-teboroxime

Kits for the preparation of Tc-99m-teboroxime were supplied in a lyophilized form by Squibb Diagnostics, Princeton, NJ, USA. A vial of teboroxime was reconstituted by the addition of 25 mCi of Tc-99m-pertechnetate. The vial was then heated for 15 min at 100°C using a heating block. After cooling to room temperature, paper chromatography was performed to determine the percentage of soluble contaminants and reduced hydrolyzed technetium. Whatman 31 ET chromatography strips (1.3 × 11 cm) and two individual mobile-phase solvent systems were used to determine the radiochemical purity of the prepared product. The developed chromatographs were air-dried and counted. The results indicated that radiochemical purity was 94.0% ± 0.4%. The compound was stored at room temperature until use, which was within 6 h of preparation. Just prior to injection, a volume of the vial with 5 mCi of activity was withdrawn into a lead-shielded syringe.

### Experimental protocol

**Figure 1:**
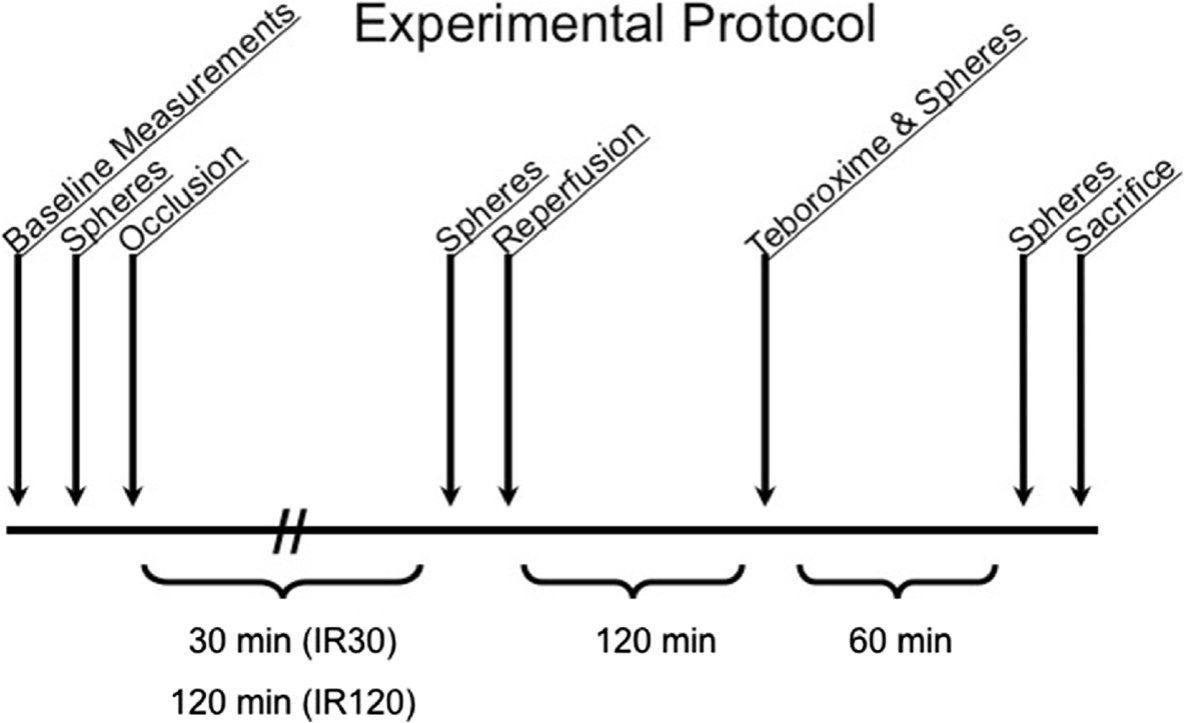
**Experimental protocol.** The IR30 group underwent 30 min of LCx occlusion followed by 120 min of reperfusion followed by Tc-99m-teboroxime administration. The IR120 group underwent 120 min of LCx occlusion followed by 120 min of reperfusion followed by Tc-99m-teboroxime administration. IR30, ischemia-reperfusion, 30-min occlusion group; IR120, ischemia reperfusion, 120-min occlusion group.

Figure 1 illustrates the experimental protocol. Baseline hemodynamic measurements were recorded during a 15-min period following instrumentation in all 13 dogs. Following the baseline period, microspheres were injected to determine regional myocardial blood flows as described below. The snare occluder on the LCx was then tightened in order to provide complete occlusion. A second microsphere blood flow measurement was made following occlusion to document the absence of coronary flow.

Six dogs undergoing mild injury received 30 min of occlusion followed by 120 min of reperfusion (IR30). Seven dogs undergoing severe injury received 120 min of occlusion followed by 120 min of reperfusion (IR120). Five mCi of Tc-99m-teboroxime were then injected into the left femoral vein in all 13 dogs. We elected to continue reperfusion for 120 min before injecting the tracer since myocardial injury is progressive for several hours after the onset of reperfusion [14]. Another microsphere blood flow determination was simultaneously performed. A cardiac output determination using the thermodilution technique was also made at this time.

To measure blood Tc-99m activity, 1.0 ml serial arterial blood samples were collected at 30-s intervals during the first 2 min and subsequently at 2, 4, 6, 8, 10, 20, 30, 60, and 120 min after injection. At the end of 1 h, a final myocardial blood flow determination was made by injecting microspheres into the left atrium, and a second cardiac output determination was made using the thermodilution technique. The dogs were then euthanized.

We assessed myocardial blood flow using the microsphere technique as has been previously described [5]. Briefly, two to three million 11-μm radiolabeled microspheres were injected into the left atrium. The microspheres were labeled with either Sn-113, Ru-103, Nb-95, or Sc-46. The order in which the microspheres were injected was balanced across experiments. Microsphere reference blood collection was begun 10 s prior to each microsphere injection and continued for 2 min following the injection.

### Post-mortem *ex vivo* analysis

Following euthanasia, the LCx was completely re-occluded, and the heart was infused with Evans blue dye to delineate the area at risk. The heart was then removed from the chest cavity and the atria and great vessels were excised. The left ventricle was cut into four 1-cm short-axis slices. The slices were then stained with triphenyltetrazolium chloride and photographed as described below. The LAD and LCx territories were then each subdivided into 24 pieces. The serial blood samples and myocardial tissue samples were counted for 3 min each in the gamma well counter (Model 1282, LKB Instruments, Gaithersburg, MD, USA) with a window setting of 120 to 160 keV to detect Tc-99m activity. These data were corrected for background and radioactive decay. The following day, the microsphere reference blood samples and the same myocardial tissue samples were again counted for 5 min each. Appropriate window settings were chosen for each microsphere isotope (Sn-113 was counted within a 350- to 435-keV window, Ru-103 within a 450- to 550-keV window, Nb-95 within a 660- to 800-keV window, and Sc-46 within a 810- to 1,200-keV window). Proprietary software was used to correct for both background and spillover activity from one window into another and to calculate regional myocardial blood flow. Regional myocardial blood flow was expressed as ml/min/g of tissue as calculated from the microsphere count data and tissue sample weights. Myocardial blood flow ratios were calculated by dividing the flow in the IRZ region by the flow in the NZ region.

### *Ex vivo* myocardial viability assessment

After euthanasia, the left ventricle was sectioned into four short-axis slices. Triphenyltetrazolium chloride (TTC) staining was then performed on the four slices to determine myocardial tissue viability. The four slices were incubated in TTC Tris solution with pH 7.8 at 37°C for 15 min. The TTC-stained myocardial slices were photographed and quantitatively analyzed using video densitometry software (SigmaScan, Jandel, CA, USA) to calculate the percentage of viable myocardium.

### Data analysis and statistical methods

The first blood sample in each experiment was discarded, as the activity of this sample was lower than that of the 30-s sample due to inadequate time for complete mixing of Tc-99m-teboroxime in the blood pool. Background- and decay-corrected serial blood sample data were normalized to the percent activity at 30 s, and activity over time curves were modeled using nonlinear regression analysis (Systat, Inc., Evanston, IL, USA).

Well counter-determined myocardial tissue Tc-99m activity was corrected for weight for the 24 pieces of normal myocardium and the 24 pieces of ischemic-reperfused myocardium. Normal zone data were combined for the six dogs in the IR30 group as well as for the seven dogs in the IR120 group. Ischemia-reperfusion zone data were combined for the six dogs in the IR30 group as well as for the seven dogs in the IR120 group.

All results were expressed as mean ± standard error of the mean (SEM). Differences in continuous variables between groups were assessed using a one-way repeated measures analysis of variance. *Post hoc* comparisons were made using Student's *t* test with correction for multiple comparisons via the Bonferroni procedure (Crunch Software Corp., Oakland, CA, USA). Within-group comparisons were made using a paired Student's *t* test. *p* values less than 0.05 were considered significant.

## Results

### Hemodynamic data

Table 1 shows complete hemodynamic data for all 13 dogs. There were no significant differences in mean arterial pressure (MAP) between the IR30 and IR120 groups at baseline. During occlusion, both the IR30 and IR120 groups had significant reductions in MAP as compared to their respective baseline values. At the time of Tc-99m-teboroxime administration and at the end of the experiment, the IR120 group continued to have significantly lower MAP as compared with both baseline values and the IR30 group.

**Table 1:** Hemodynamic parameters

	**Baseline**	**Post-occlusion**	**Tc injection**	**Final**
MAP (mmHg)				
IR30	104.3 ± 2.4	91.0 ± 7.5†	102.0 ± 1.7	103.3 ± 1.6
IR120	102.6 ± 3.0	89.6 ± 4.1†	91.7 ± 3.5*†	92.6 ± 3.7*†
HR (bpm)				
IR30	117.5 ± 4.0	125.5 ± 6.5	110.8 ± 4.9	103.7 ± 3.2
IR120	125.7 ± 9.8	103.7 ± 3.3*†	96.6 ± 5.0†	91.0 ± 2.5*†
CO (ml/min)				
IR30	1.8 ± 0.4	1.8 ± 0.4	2.1 ± 0.4	2.4 ± 0.3
IR120	1.8 ± 0.1	1.7 ± 0.1	2.5 ± 0.2	2.1 ± 0.1
DP (mmHg)				
IR30	102.0 ± 13.0	26.6 ± 0.7†	92.0 ± 4.2	93.7 ± 3.7
IR120	98.2 ± 3.6	24.2 ± 0.7†	92.8 ± 7.2	96.3 ± 8.4
LAP (mmHg)				
IR30	4.3 ± 0.6	8.0 ± 1.2†	6.1 ± 0.6†	8.1 ± 0.3†
IR120	4.5 ± 0.3	12.7 ± 1.1†	6.5 ± 1.1†	6.2 ± 0.7†

*p < 0.05 compared to normal zone; †*p* < 0.05 compared to baseline. CO, cardiac output; DP, distal left circumflex coronary artery pressure; IR30, 30-min occlusion (*n* = 6); HR, heart rate; IR120, 120-min occlusion (*n* = 7); LAP, left atrial pressure; MAP, mean arterial pressure.

Heart rate was not significantly different between the two experimental groups at baseline. At the time of occlusion, Tc-99m-teboroxime administration, and at the end of the experiment, the IR120 group had significantly lower heart rate as compared with baseline values. At the time of occlusion and at the end of the experiment, the IR120 group had significantly lower heart rate as compared with the IR30 group, but this did not have a significant effect on cardiac output, which was not significantly different between the two groups at any time during the experiment.

Distal LCx arterial pressure was not significantly different between the two experimental groups at baseline. At the time of occlusion, both the IR30 and IR120 groups had significantly lower distal LCx pressure as compared with their respective baseline values. At the time of Tc-99m-teboroxime administration and at the end of the experiment, distal pressure was not significantly different from baseline values for either group.

Left atrial pressure was not significantly different between the two experimental groups at baseline. Beginning at the time of LCx occlusion and continuing through the end of the experiment, both the IR30 and IR120 groups had significant rises in left atrial pressure as compared with baseline values, but there were no significant differences between the two groups.

### Regional myocardial function

Percent left ventricular wall thickening was calculated from sonomicrometer-determined end systolic and end diastolic wall thicknesses at baseline, LCx occlusion, Tc-99m-teboroxime administration, and at the end of the experiment. These data are presented in Table 2. Both the IR30 and IR120 groups demonstrated decreases in percent wall thickening in the IRZ as compared with the NZ following LCx occlusion. In the IR30 group, percent wall thickening in the IRZ recovered as compared with the NZ during the reperfusion phase, suggesting that most of the IRZ suffered reversible injury in the IR30 group. In the IR120 group, percent wall thickening in the IRZ remained decreased as compared with the NZ during the reperfusion phase and through the end of the experiment, suggesting that most of the IRZ suffered irreversible injury in this group.

**Table 2:** Wall thickening fraction

	**Initial**	**Post-occlusion**	**Tc injection**	**Final**
IR30				
Normal zone	22.3 ± 3.8	20.6 ± 3.6	22.7 ± 5.1	20.3 ± 3.2
IR zone	22.7 ± 4.3	16.3 ± 1.6*†	21.6 ± 5.3	20.8 ± 3.9
IR120				
Normal zone	18.6 ± 1.5	19.1 ± 1.5	19.5 ± 2.2	20.2 ± 2.0
IR zone	18.2 ± 1.3	15.5 ± 1.0*†	16.0 ± 1.5	14.7 ± 1.1*†

*p < 0.05 as compared to IR30; †*p* < 0.05 as compared to baseline. IR, ischemia-reperfusion; IR30, 30-min occlusion (*n* = 6); IR120, 120-min occlusion (*n* = 7).

### Area at risk and infarcted area

**Figure 2:**
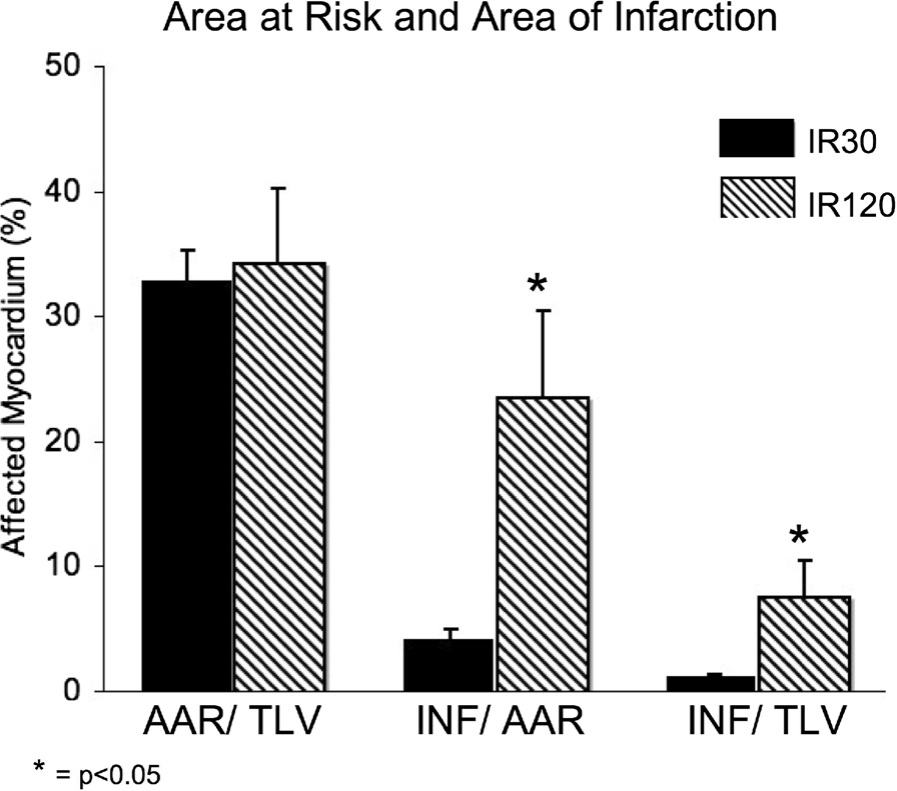
**Myocardial area at risk and infarcted area.** This figure illustrates the percentage of the total left ventricle at risk as determined by Evans blue dye, the percentage of the area at risk that ultimately infarcted as determined by TTC staining, and the percentage of the total left ventricle that ultimately infarcted as determined by TTC staining. *p < 0.05 as compared to IR30. AAR, area at risk; INF, infarcted area; IR30, ischemia-reperfusion, 30-min occlusion group; IR120, ischemia-reperfusion, 120-min occlusion group; TLV, total left ventricle.

**Figure 3:**
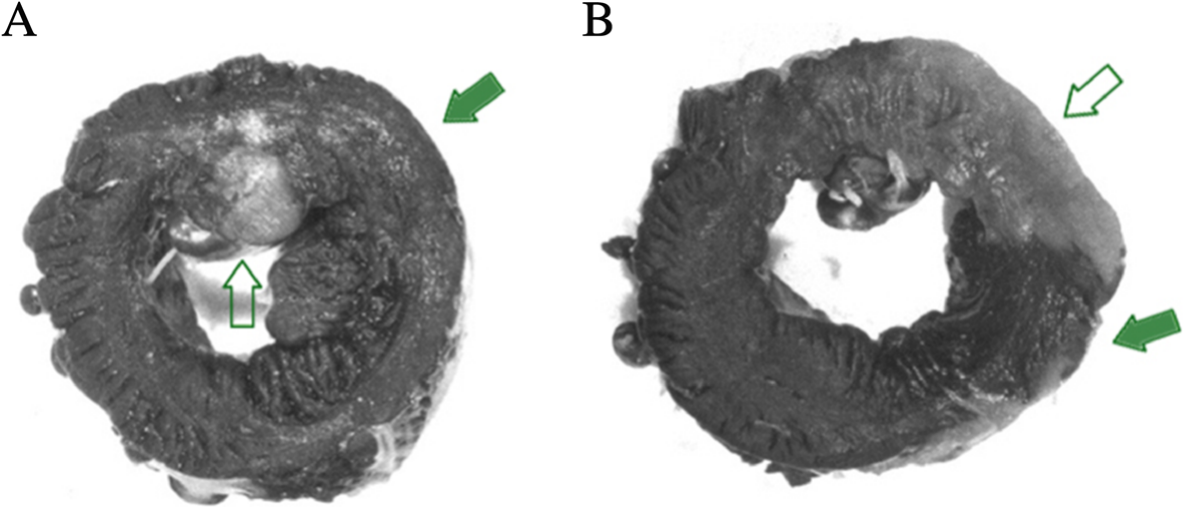
**Examples of TTC staining/Evans blue dye images.** A dog with a small infarct **(A)** and a dog with a large infarct **(B)**. The open arrows point to the infarct zones. The solid arrows point to the Evans blue-stained area at risk.

Figure 2 shows the area at risk as determined by Evans blue staining and the percent infarcted myocardium as determined by TTC staining for the two experimental groups. The area at risk expressed as a percentage of total left ventricular myocardium was 32.7% ± 2.5% for the IR30 group and 34.2% ± 6.0% for the IR120 group (*p* = ns). The infarcted area as a percentage of the area at risk was 4.0% ± 0.9% for the IR30 group, compared with 23.5% ± 6.9% for the IR120 group (*p* < 0.05). The infarcted area as a percentage of total left ventricular myocardium was 1.1% ± 0.3% for the IR30 group and 7.5% ± 2.9% for the IR120 group (*p* < 0.05). Figure 3 shows the TTC/Evans blue images from representative dogs.

### Myocardial blood flow ratio data

**Figure 4:**
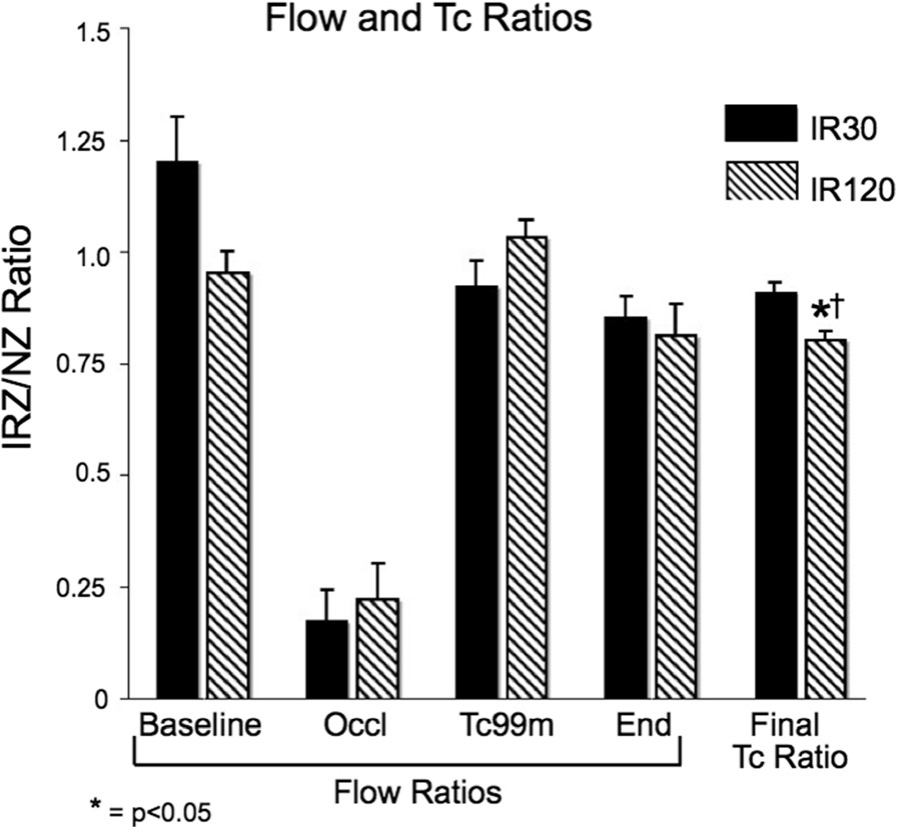
**Myocardial blood flow and Tc-99m ratios.** Myocardial blood flow ratios (IRZ/NZ) were determined by microspheres for both study groups at four time points. Gamma well counter-determined myocardial tissue Tc-99m ratios (IRZ/NZ) at the end of 1 h of clearance are also shown. *p < 0.05 as compared to IR30 final Tc ratio; †*p* < 0.05 as compared to IR120 Tc-99m flow ratio. End, 1 h after tracer administration; IR30, ischemia-reperfusion, 30-min occlusion group; IR120, ischemia reperfusion, 120-min occlusion group; IRZ, ischemia-reperfusion zone; NZ, normal zone; Occl, time of LCx occlusion; Tc-99m, time of Tc-99m-teboroxime administration.

Regional myocardial blood flows were determined by the radiolabeled microsphere technique as described above, and flow ratios were calculated for the two experimental groups at four time points (Figure 4). During the control period, flow ratios (LCx/LAD) were near unity and were not significantly different between the two groups. Following LCx occlusion, both groups had significantly reduced flow ratios as compared to their respective baseline values. Following reperfusion and at the end of the study, both groups had flow ratios that were not significantly different as compared to their respective baseline values.

### Gamma well counted tissue ratios

Myocardial tissue in the IR120 group demonstrated significantly lower gamma well counted Tc-99m ratio (IRZ/NZ) at the end of 1 h (0.80 ± 0.01) as compared to the IR30 group (0.93 ± 0.01) (*p* < 0.05) (Figure 3). This suggests minimal differential clearance of Tc-99m-teboroxime in the IR30 group, which sustained mild injury, and moderate differential clearance of Tc-99m-teboroxime in the IR120 group, which sustained severe injury. Furthermore, the IR120 group gamma well counted Tc-99m ratio (0.80 ± 0.01) was significantly lower than the IR120 group flow ratio at the time of Tc-99m-teboroxime administration (1.05 ± 0.06) (*p* < 0.05). This supports the finding of moderate differential clearance of Tc-99m-teboroxime in the IR120 group.

### Blood clearance kinetics

**Figure 5:**
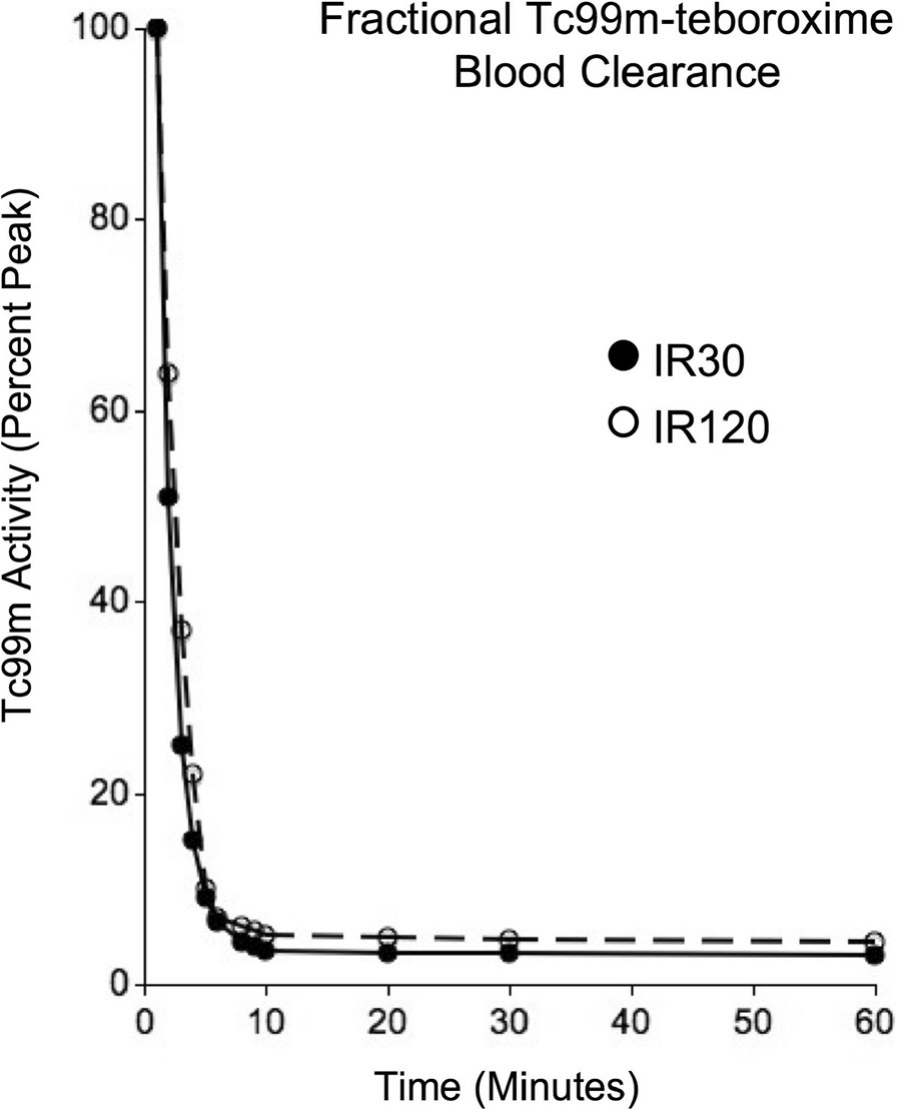
**Fractional Tc-99m-teboroxime blood clearance curves.** This figure displays tracer activity at each time point normalized to peak activity. IR30, ischemia-reperfusion, 30-min occlusion group; IR120, ischemia reperfusion, 120-min occlusion group.

Figure 5 shows mean Tc-99m-teboroxime blood clearance data for the two experimental groups. The activity at each time point was normalized to peak activity. In both groups, Tc-99m-teboroxime cleared rapidly from the blood during the first 5 min, with slower clearance over the remaining period of study.

## Discussion

Tc-99m-teboroxime has demonstrated a number of unique and favorable characteristics for clinical myocardial imaging compared with other available imaging agents. Myocardial extraction is exceptionally high [15],[16] and is relatively insensitive to metabolic impairment [11]. The agent is an excellent flow marker, even at high flow rates [3],[4],[6]. Additionally, rapid clearance kinetics allow for serial studies and shorter stress/rest protocols [17]. Finally, differential clearance has been observed in animal models of resting ischemia [4],[5] and dypyridamole-mediated stress in the setting of coronary occlusion [18], as well as in patients with stable coronary artery disease [7],[9],[10]. Studies from our laboratory have suggested the potential utility of differential clearance in determining the severity of ischemia [5].

While several studies have shown that Tc-99m-teboroxime uptake is a flow marker independent of myocyte viability [19]–[25], relatively little is known regarding the effects of altered myocyte viability on tracer retention. Furthermore, the mechanism of uptake for Tc-99m-teboroxime is unclear. Maublant et al. used cultured myocytes to show that Tc-99m-teboroxime localizes primarily to the myocyte membrane [11]. Our laboratory in turn demonstrated accelerated clearance of the tracer following myocyte membrane injury due to Triton-X 100 in an isolated perfused rat heart model [12]. Abraham and co-workers used a porcine model of acute myocardial infarction with reperfusion to demonstrate that viable myocytes are required for retention of Tc-99m-teboroxime [13]. Maublant et al. demonstrated both high extraction in cultured rat cardiac myocytes and lack of response to the presence of metabolic inhibitors shown to accelerate sestamibi clearance [26]. Rosenspire et al. showed rapid, high-affinity binding to rat plasma proteins and blood cells both *in vitro* and *in vivo*[27]. Dahlberg et al. demonstrated that binding to blood components reduced myocardial extraction and increased the rate of clearance of Tc-99m-teboroxime in blood-perfused rabbit hearts [28]. Pirro et al. also found high first-pass extraction in rat brain and cultured cells with low transendothelial permeability [29]. Studies using another neutrally charged cardiac radiotracer, 99m-Tc-NOET, have shown similar results [30],[31]. Taken together, these results indicate that Tc-99m-teboroxime has affinity for membranes in a variety of cell types due to its molecular characteristics which include neutral charge, high lipophilicity, and high hydrophobicity. Furthermore, it appears that the mechanism of uptake for neutrally charged cardiac radiotracers is not the same as that for positively charged molecules such as thallium and sestamibi.

In the present study, we hypothesized that coronary occlusion followed by reperfusion would lead to more rapid myocardial clearance of Tc-99m-teboroxime due to myocyte membrane damage resulting from both ischemia and reperfusion injury. We used an occlusion-reperfusion protocol with varying durations of occlusion to produce mild (IR30) and severe (IR120) myocardial injury, and demonstrated that Tc-99m-teboroxime clearance kinetics can differentiate between the two.

### Hemodynamics, blood flow, and contractility

The pattern of hemodynamic alterations observed was consistent with stenosis of a major coronary artery and included decreased systemic arterial pressure, decreased coronary artery pressure distal to the stenosis, slightly elevated left atrial pressure, and decreased heart rate. The mean arterial pressure recovered in the mild injury group (IR30), but persisted in the severe injury group (IR120). However, there was no difference in cardiac output between the groups ensuring that reduced heart rate and arterial pressure did not account for the observed differences in tracer kinetics. Tc-99m-teboroxime injection produced no observable hemodynamic changes in these experiments.

Prior to LCx occlusion, there were no differences in absolute microsphere-determined regional myocardial blood flows between the two groups. As expected, absolute blood flow was significantly reduced during LCx occlusion and then normalized after reperfusion in both experimental groups.

Wall thickening in the ischemia-reperfusion zone (IRZ) decreased as expected in both groups during LCx occlusion. Wall thickening in the IRZ normalized following reperfusion in the IR30 group, but was persistently decreased in the IR120 group consistent with a greater degree of injury.

### Area at risk and infarct size

The area at risk during LCx occlusion as determined by Evans blue dye was not significantly different between the two experimental groups. As expected, the TTC-determined infarct size was significantly greater in the IR120 group. Infarct size was only 1% of the total LV area for the IR30 group.

### Tc-99m-teboroxime differential clearance

Previous studies in our laboratory using a model of resting ischemia without reperfusion and without injury demonstrated significantly reduced clearance in ischemic zones resulting exclusively from reduced flow [32]. In a perfused rat model, our laboratory showed markedly accelerated clearance in the setting of membrane injury due to Triton-X 100, a membrane detergent [12].

In the current study using canine models of mild and severe injury, the final gamma well counter-determined Tc-99m-tissue ratios (IRZ/NZ region) were less than unity for both the IR30 and IR120 groups, suggesting differential clearance in both groups. However, the Tc-99m-teboroxime ratio (IRZ/NZ) was significantly lower for the IR120 group compared to the IR30 group. This suggests more rapid IRZ tracer clearance in the severe injury group (IR120) as compared to the mild injury group (IR30). Furthermore, the final gamma well counter-determined Tc-99m ratio (IRZ/NZ) was significantly less than the microsphere-determined blood flow ratio (IRZ/NZ) at the time of tracer administration in the severe injury group (IR120), confirming differential clearance of the tracer in this group.

### Blood clearance kinetics

Tc-99m-teboroxime blood clearance was rapid with 90% of the radiotracer being cleared in the first 10 min, which was in agreement with the previous data [32]. There was no significant difference in blood clearance between the two groups.

### Clinical implications

The current study demonstrates the potential for differentiating severe injury from both mild injury and normal myocardium using differential Tc-99m-teboroxime clearance kinetics. Fortunately, myocardial extraction from the blood pool is rapid and permits the commencement of imaging within minutes of tracer injection. However, as Tc-99m-teboroxime clearance is also rapid, fast imaging protocols are necessary. Newer solid-state SPECT imaging technology makes such acquisitions more feasible. Quantitative techniques may also be useful in detecting small differences in clearance.

## Conclusions

Tc-99m-teboroxime clearance kinetics differentiate severely injured myocardium from normal and minimally injured myocardium in a canine model of acute myocardial injury due to coronary occlusion and reperfusion. Further clinical studies are warranted to determine if rapid image acquisition technology will allow utilization of these kinetic properties in patient diagnosis.

## Competing interests

The authors declare that they have no competing interests.

## Authors’ contributions

DRO carried out the data analysis, statistical analysis, and table and figure preparation. He was primarily responsible for the preparation of the manuscript and multiple revisions. GJ and RDO were responsible for the study design and experimental protocols. They assisted DRO in the preparation of the manuscript. All authors read and approved the final manuscript.
